# Changes of Plasma FABP4, CRP, Leptin, and Chemerin Levels in relation to Different Dietary Patterns and Duodenal-Jejunal Omega Switch Surgery in Sprague–Dawley Rats

**DOI:** 10.1155/2018/2151429

**Published:** 2018-04-22

**Authors:** Dominika Stygar, Elżbieta Chełmecka, Tomasz Sawczyn, Bronisława Skrzep-Poloczek, Jakub Poloczek, Konrad Wojciech Karcz

**Affiliations:** ^1^Department of Physiology, School of Medicine with Dentistry Division in Zabrze, Medical University of Silesia, Katowice, Poland; ^2^Department of Statistics, Department of Instrumental Analysis, School of Pharmacy with the Division of Laboratory Medicine in Sosnowiec, Medical University of Silesia, Katowice, Poland; ^3^Department of Rehabilitation, 3rd Specialist Hospital, Rybnik, Poland; ^4^Clinic of General, Visceral, Transplantation and Vascular Surgery, Hospital of the Ludwig Maximilian University, Munich, Germany

## Abstract

**Background:**

Pathophysiological links between inflammation, obesity, and adipokines can be used for the treatment of metabolic dysregulation.

**Aims:**

To examine the influence of duodenal-jejunal omega switch surgery in combination with different diet patterns on plasma concentrations of fatty acid-binding protein 4 (FABP4), C-reactive protein (CRP), leptin, and chemerin.

**Methods:**

After 8 weeks on a high-fat diet (HF) or control diet (CD), rats underwent surgery. Duodenal-jejunal omega switch (DJOS) with an exclusion of one-third of intestinal length and SHAM surgery were performed. For the next 8 weeks, 50% of DJOS/SHAM animals were kept on the same diet as before (HF/DJOS/HF, HF/SHAM/HF, CD/DJOS/CD, and CD/SHAM/CD), and 50% had a changed diet (HF/DJOS/CD, HF/SHAM/CD, CD/DJOS/HF, and CD/SHAM/HF). FABP4, CRP, leptin, and chemerin were assessed using ELISA kits.

**Results:**

FABP4: significant differences between DJOS and SHAM were observed in animals maintained on CD/CD; CRP: varied between DJOS and SHAM groups maintained on HF/HF, CD/CD, and CD/HF; leptin and chemerin levels: DJOS lowered leptin and chemerin plasma levels versus SHAM, while HF/HF, CD/HF, and HF/CD significantly increased leptin and chemerin plasma levels when compared to CD/CD.

**Conclusions:**

The beneficial effect of DJOS surgery is stronger than proinflammatory conditions caused by an HF obesogenic diet.

## 1. Introduction

Systematic energy surplus with “unhealthy dietary patterns” is known to be a strong driver in the development of obesity, for which a chronic low-grade inflammatory condition called metabolically triggered inflammation, metainflammation, or parainflammation is characteristic [1, 2]. The condition of chronic inflammation if occurs in metabolically involved organs, like liver and adipose tissue, plays a main role in the development of chronic metabolic diseases, such as diabetes, and fatty liver disease [2]. Adipose tissue, aside from controlling fat mass and nutrient homeostasis, realises an inflammatory cytokine, which regulates metabolic homeostasis and the immune response. Fatty acid-binding proteins (FAPBs) are intracellular proteins known to facilitate lipid-mediated processes in cells [3]. So far, 9 types of FAPBs have been described and named in relation to the organ/tissue expression [4]. FABP4, expressed by adipocytes and macrophages, plays a key role in regulating systemic metabolism. It is an important mediator of inflammatory processes and metabolic syndrome [1]. The low-grade systemic inflammation is as well characterised by C-reactive protein (CRP). CRP is produced in the liver on the binding of proinflammatory cytokines and is associated with obesity [5]. Leptin is an adipose-derived cytokine, which shows appetite-suppressant acting mediated through hypothalamic signaling. Leptin-resistant conditions, characteristic for obesity, lead to a loss of hypothalamus control of appetite and feeding behavior, exacerbating the already excessive body weight gain [6]. Leptin stimulates the gene expression of proinflammatory cytokines such as tumor necrosis factor alpha (TNF-*α*), interleukin 1 (IL-1), and interleukin (IL-6), improves phagocytosis by regulating oxidative stress, and acts on immune cells [7–11]. Chemerin, also called retinoic acid receptor responder 2 or tazarotene-induced gene 2, has been found to be highly expressed in adipose tissue. Plasma total chemerin concentrations are positively associated with obesity, metabolic syndrome, and inflammation [12]. Chemerin merges obesity with inflammation inactivating the orphan G-protein coupled receptor chemokine-like receptor 1 (CMKLR1, ChemR23) which is characteristic for cells of the innate immune system [12–15]. Bariatric surgery is one of the most efficient treatments for long-term weight loss and its long-term maintenance [16]. A systematic review of long-term follow-ups after bariatric surgery shows that this is one of the most efficient treatments of obesity, which helps to reduce body weight by up to 60% during the first two years after surgery, and gives about a 38% reduction of comorbidities in studied individuals [17]. Duodenal-jejunal omega switch (DJOS) is a type of bariatric surgery, with proximal loop duodeno-enterostomy, which bypasses the foregut (foregut theory), and allows for direct hindgut stimulation (hindgut theory) [18, 19]. The advantage of DJOS is a bypass-like procedure, where the pylorus of the patients is saved. This modification prevents patients from symptoms characteristic for postgastrectomy conditions such as dumping, diarrhoea, and dyspepsia [20, 21]. DJOS is a relatively new technique, thus an animal model, for exploring the physiological long-term effects of this procedure, is still needed [20, 22].

A pathophysiological link between dietary patterns, obesity, inflammation, and adipokines can be used as a potential target in therapeutic strategies for treatment of metabolic dysregulation like obesity, insulin resistance, and T2DM. Thus, the aim of the study was to measure the impact of DJOS surgery in combination with different types of dietary patterns on FABP4, leptin, chemerin, and CRP plasma levels. In this study, we decided to use an HF diet in order to induce obesity as a model close to human behaviour for the investigation of the underlying mechanisms mediating metabolic benefits of DJOS, measured by the plasma levels of selected adipokines. The experimental design of this study also includes the observations that not all patients after surgery follow the nutritional recommendations [23, 24]. Many physiological and psychological factors influence postoperative differences in weight-related outcomes, but it is known that weight regain occurs in up to 20% of patients after the surgery [25–27]. Thus, the selected study groups differ in terms of diet used before and after surgery. We assumed that after a bariatric operation, one might switch from a regular diet to an HF and from an HF to a regular diet. Then, we assessed the effect of duodenal-jejunal omega switch surgery in combination with CD, and an HF diet, before and after surgery, on the plasma levels of selected adipokines.

## 2. Materials and Methods

### 2.1. Animals and Diets

This individual study is based on experimental design applied and described in an earlier work by Stygar et al. [28]. Seven-week-old, male Sprague–Dawley rats (Charles River Laboratories Inc., Wilmington, MA) were kept in 12 h light-dark cycles at 22°C and 40–60% humidity. Environmental enrichment was provided, and all rats had free access to water and food. The composition of control diet (CD) was (Provimi Kliba AG, Kaiseraugst, Switzerland) 24% protein, 4.9% fat, 7% crude ashes, 4.7% crude fiber, lysine (13.6 g/kg), calcium (12 g/kg), methionine (4.5 g/kg), and phosphorus (8.3 g/kg). The animals from the control group were kept on CD for the period of two months, before and after surgery. Obesity was induced by keeping the animals on a high-fat diet (HF; 23.0 kJ/g; 59% fat, 27% carbohydrate, and 14% protein (EF RAT [E15744] Ssniff Spezialdiäten GmbH, Soest, Germany)) for the period of two months before and after surgery. All rats fasted overnight before surgery.

## 3. Experimental Design

**Figure 1:**
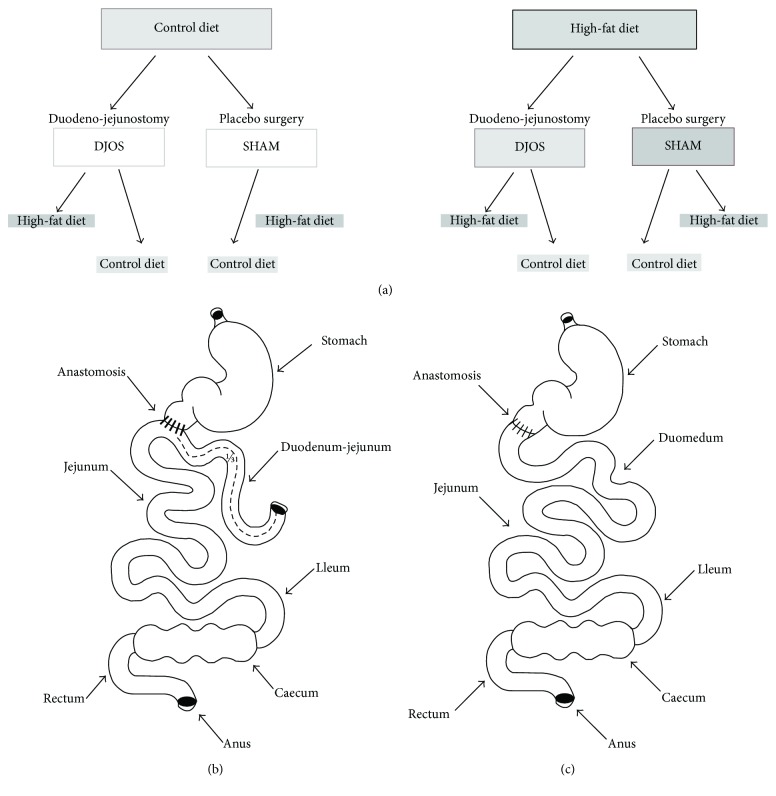
(a) Scheme of experimental groups. (b) Schematic illustration of DJOS. (c) SHAM surgery.

After one week of acclimatisation, the animals were assigned to the experimental dietary patterns HF groups (*n* = 28) and CD (*n* = 28). The total duration of the experiment was 16 weeks. The animals were kept on selected diets for the period of 8 weeks before and 8 weeks after the DJOS and SHAM surgery. The first part of the protocol, before the surgery, included 8 weeks of maintenance of animals on selected diets. After this time, both groups (CD and HF) were divided into two subgroups, which underwent different types of surgery: 50% of rats underwent DJOS (14 animals) and 50% underwent SHAM surgery, which is a control type of surgery (14 animals, Figure 1(a)).

In the second part of the experiment, after the surgery, 50% of DJOS/SHAM animals were kept on the same diet as before (HF/DJOS/HF, HF/SHAM/HF, CD/DJOS/CD, and CD/SHAM/CD), and 50% had changed the diet (HF/DJOS/CD, HF/SHAM/CD, CD/DJOS/HF, and CD/SHAM/HF; Figure 1(a)). The “3Rs” for the ethical treatment of animals was followed in the study [29]. In the HF/SHAM/CD subgroup, 6 out of 7 rats survived till the end of the experiment, while in the rest of subgroups, the survival was 100%.

## 4. Experimental Procedures

A DJOS was performed according to Karcz et al. methodology [22], described in the aforementioned study [28]. To perform DJOS, the animals were anaesthetized with 2% isoflurane (AbbVie Deutschland GmbH & Co. KG, Ludwigshafen, Germany) and oxygen flow at 2 l/min under spontaneous breathing. Analgesia with xylazine (5 mg/kg, ip; Xylapan, Vetoquinol Biovet, Poland) and antibiotic prophylaxis with gentamicin were applied. In order to gain abdominal access, a midline incision of 3-4 cm was performed, and the total length of the small intestine was determined (Figure 1(b)). The stomach was separated from the duodenum at the point just below the pylorus, and the position of anastomosis was defined at 1/3 of the total small bowel length. The jejunum was anastomosed via end-to-side duodeno-enterostomy in order to restore the physiological conduit of the food passage, excluding the duodenum and parts of the small intestine. The remaining duodenal stump was closed using PDS 6/0 (Ethicon). Mesenteric openings were closed with PDS 6/0 (Ethicon).

In the SHAM-operated animals, reanastomosis of the gastrointestinal tract was performed at the corresponding sites where enterotomies were performed for the duodenojejunostomy, thereby maintaining continuity of the food passage through the bowel (Figure 1(c)). For DJOS and SHAM protocols, postoperative analgesia was performed using carprofen (4 mg/kg, sc; Rimadyl, Pfizer, Switzerland) for 3 consecutive days after the surgery.

### 4.1. Tissue Collection and Assay Identification

At the end of the 8th week after surgery, corresponding to the 16th week of the experiment, blood samples for adipokines and CRP measurements were collected from the abdominal aorta, using tubes containing 10 *μ*l EDTA (Sigma-Aldrich, St. Louis, MO). After centrifugation at 4000 rpm for 10 minutes at 4°C, plasma samples were collected, snap-frozen in liquid nitrogen, and stored at −80°C until analyses were performed. Adipokines, including FABP4, leptin, chemerin, and CRP, were assessed in duplicate by using sandwich ELISA kits (Cloud-Clone Corp., Katy, TX). All experimental procedures were approved by the Ethical Committee for Animal Experimentation (58/2014).

## 5. Statistical Analysis

Statistical analysis was performed using STATISTICA 12.5 PL (StatSoft, Cracow, Poland). Statistical significance was set at a *p* value below 0.05. All tests were two-tailed. Interval data were expressed as mean value ± standard deviation in the case of normal distribution, or as median/lower–upper quartile range in the case of data with skewed or nonnormal distribution. Distribution of variables was evaluated by the Shapiro-Wilk test and the quantile-quantile plot. The homogeneity of variances was assessed by the Levene test. For comparison of data, the two-way parametric ANOVA and post hoc contrast analysis or nonparametric Kruskal-Wallis test or Mann–Whitney *U* test were used. In case of skewed data distribution, logarithmic transformation was performed before analysis.

## 6. Results

The results of body weight change after DJOS and SHAM surgery in all experimental groups were previously presented by Stygar et al. [28]. Plasma concentrations of FABP4, CRP, leptin, and chemerin in DJOS and SHAM-operated groups after long-term maintenance on HF and CD and mixed HF/CD and CD/HF eating patterns are shown in Table 1.

Table 2 presents results of multiple comparisons in contrast analysis of DJOS and SHAM-operated groups in relation to diet used before and after surgery. Column one presents a comparison between DJOS and SHAM surgery associated with different diets, column two shows comparisons between dietary groups of DJOS operated animals, and column three presents comparisons between dietary groups of SHAM-operated animals.

### 6.1. Plasma Concentrations of FABP4

**Figure 2:**
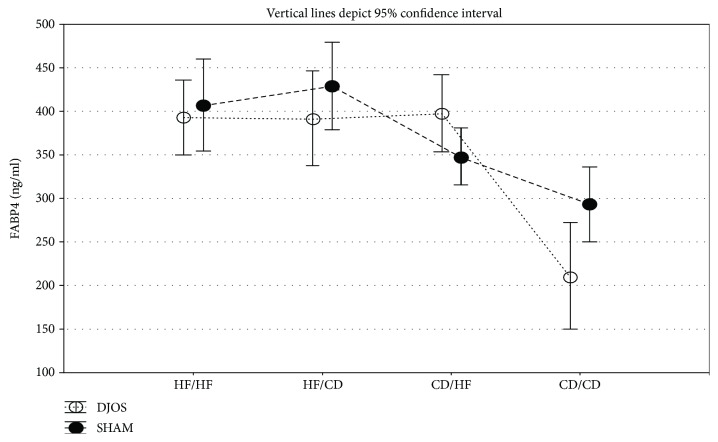
Mean values of FABP4 (ng/ml) plasma levels in four groups subjected to different dietary patterns, according to the DJOS and SHAM operation type. Statistical significance was set at *p* < 0.05. Vertical lines depict 95% confidence interval. DJOS: duodenal-jejunal omega switch surgery; HF: high-fat diet; CD: control diet; HF/HF, CD/HF, HF/CD, CD/CD: type of diet 8 weeks before/8 weeks after surgery.

The type of diet strongly influenced the FABP4 plasma levels both in animals from the DJOS and SHAM groups. Significant differences between DJOS and SHAM groups were observed only in animals maintained on CD diet before and after the surgery (*p* < 0.01; Figure 2, Tables 1 and 2).

In animals after DJOS, approximately two times lower plasma level of FABP4 was observed in the CD/CD group compared with the HF/HF, HF/CD, and CD/HF groups (*p* < 0.001; Figure 2, Tables 1 and 2).

In the control groups, plasma concentrations of FABP4 in rats subjected to HF diet before and after the surgery (HF/HF) were significantly higher than those in the groups maintained on CD before the surgery (CD/HF *p* < 0.05 and CD/CD *p* < 0.001; Figure 2, Tables 1 and 2). In addition, the change from HF to CD diet significantly increased FABP4 level than the change from CD to HF and CD/CD groups (*p* < 0.01 and *p* < 0.001, resp.; Figure 2, Tables 1 and 2).

### 6.2. Plasma Concentrations of CRP

**Figure 3:**
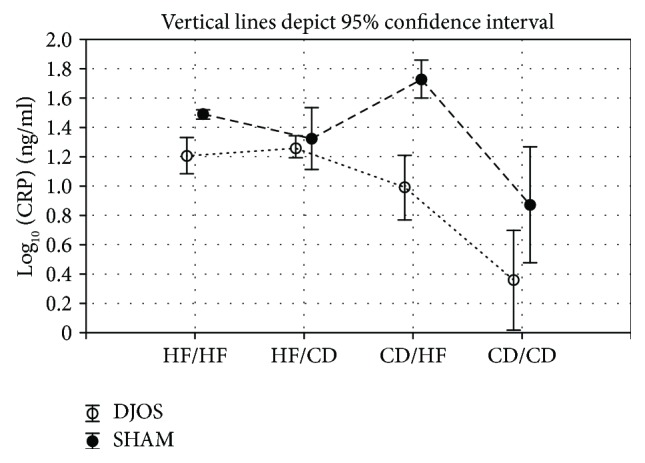
Mean values of CRP (ng/ml) plasma levels in four groups of different dietary patterns, according to the DJOS and SHAM operation type. Statistical significance was set at *p* < 0.05. Vertical lines depict 95% confidence interval. DJOS: duodenal-jejunal omega switch surgery; HF: high-fat diet; CD: control diet; HF/HF, CD/HF, HF/CD, CD/CD: type of diet 8 weeks before/8 weeks after surgery.

The CRP concentrations were consistently lower after DJOS compared with SHAM surgery regardless of dietary pattern. CRP plasma concentrations varied between DJOS and SHAM groups maintained on HF diet before and after the surgery (*p* < 0.001), CD diet (*p* < 0.05), and mixed CD/HF diet (*p* < 0.01; Figure 3, Tables 1 and 2).

For both types of surgery, the values of CRP did not differ between selected experimental groups (Tables 1 and 2).

### 6.3. Plasma Concentrations of Leptin

**Figure 4:**
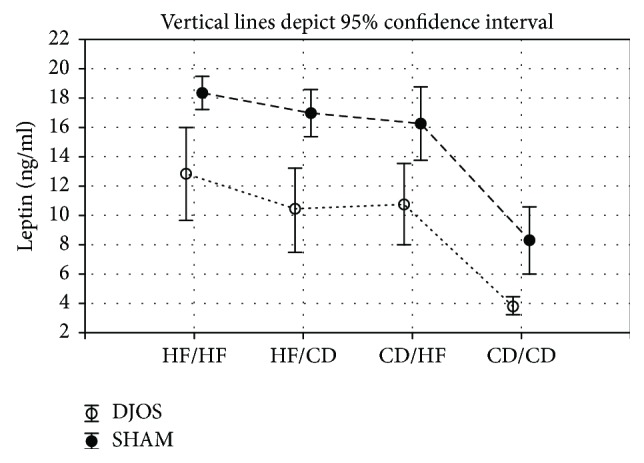
Mean values of leptin (ng/ml) plasma levels in four groups of different dietary patterns, according to the DJOS and SHAM operation type. Statistical significance was set at *p* < 0.05. Vertical lines depict 95% confidence interval. DJOS: duodenal-jejunal omega switch surgery; HF: high-fat diet; CD: control diet; HF/HF, CD/HF, HF/CD, CD/CD: type of diet 8 weeks before/8 weeks after surgery.

DJOS surgery significantly lowered the leptin plasma level in comparison to SHAM surgery despite the type of diet applied before and after surgery (*p* < 0.001 for all; Figure 4, Tables 1 and 2).

HF diet significantly increased leptin level when compared to the CD/CD group in DJOS-operated animals (*p* < 0.001; Figure 4, Table 2). The maintenance of animals on different types of diet before and after surgery increased the leptin plasma level but did not reduce the positive effect of DJOS. The CD/HF and HF/CD diets significantly increased leptin plasma levels when compared to the CD/CD group (*p* < 0.001, resp.; Figure 4, Table 2).

Also in the SHAM-operated animals, the HF diet significantly increased the leptin level in comparison to the control group (*p* < 0.001, resp.; Figure 4, Table 2).

### 6.4. Plasma Concentrations of Chemerin

**Figure 5:**
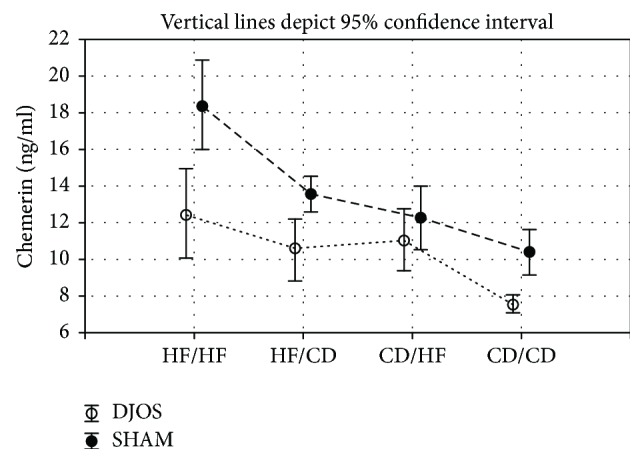
Mean values of chemerin (ng/ml) plasma levels in four groups of different dietary patterns, according to the DJOS and SHAM operation type. Statistical significance was set at *p* < 0.05. Vertical lines depict 95% confidence interval. DJOS: duodenal-jejunal omega switch surgery; HF: high-fat diet; CD: control diet; HF/HF, CD/HF, HF/CD, CD/CD: type of diet 8 weeks before/8 weeks after surgery.

DJOS surgery significantly lowered the chemerin plasma levels when compared to SHAM surgery for all analysed groups, except CD/HF (*p* < 0.001, 0.01, and 0.01, resp.; Figure 5, Tables 1 and 2).

After DJOS surgery, the highest level of chemerin was observed in the HF/HF group in comparison with all other analysed diet combinations, and it was significantly higher when compared to the CD/CD group (*p* < 0.001; Figure 5, Table 2). The lowest level of chemerin was detected for the CD/CD group, and this value was significantly different from all other groups (*p* < 0.001, 0.01, and 0.001, resp.; Figure 5, Table 2).

The type of food given to animals before and after SHAM surgery influenced the chemerin plasma level in SHAM-operated animals. The HF/HF diet changed the chemerin plasma profile in comparison with all other analysed groups (*p* < 0.001, resp.; Figure 5, Table 2). Also, in group CD/CD, the chemerin plasma level was significantly lower when compared to HF/CD groups (*p* < 0.01; Figure 5, Table 2).

## 7. Discussion

Patients with severe obesity need to adopt new dietary patterns after metabolic surgery in order to achieve long-term results. Our understanding of the effect of bariatric surgery on the systematic metabolism is still incomplete. It is not possible to distinguish between the physiological effects of dietary changes, reduced food consumption, and the direct effects of metabolic surgery per se [30].

Our present study shows the influence of dietary patterns applied before and after DJOS and SHAM surgery on the plasma FABP4 concentration. Despite the type of surgery, in FABP4, the plasma levels were influenced by the type of dietary pattern (HF and/or CD diet). HF led to the increase in FABP4 plasma concentration, reducing the positive effect of DJOS. FABP4 is characteristic for adipose tissue and macrophages, where it regulates adipocyte fatty-acid uptake and lipogenesis, and also influences cholesterol accumulation. It also delivers lipids to nuclear receptors, stimulating nuclear transcriptional patterns. In macrophages, FABP4 is known to modulate inflammatory responses by the connection to systemic inflammation and the immune system [4, 31, 32]. The elevated plasma FABP4 level is a negative prognostic factor, correlated with metabolic syndrome, insulin resistance (calculated as HOMA-IR), and mortality of patients with advanced hepatic cirrhosis and sepsis [31, 33]. Witczak et al. observed significant changes in free plasma FABP4 concentrations with time, after biliopancreatic diversion surgery. The highest level of FABP4 was observed after the 1-month follow-up and might be related to increased lipolysis after the surgery [34]. Some studies show inconsistency regarding FABP4 plasma concentrations in patients after weight loss, which may be interpreted as a normalisation of FABP4 plasma level [34–36]. In our previous study, we did not observe significant weight loss in rats maintained on an HF diet after DJOS surgery [28]. As we demonstrate here, the HF diet before and/or after both DJOS and control, SHAM surgery led to an increase in FABP4 concentration in reference to the control diet. We believe that an HF dietary pattern increases the fatty acid metabolism and is connected with upregulated FABP4 expression and secretion from the adipose tissue.

Obesity and an HF diet are associated with low-grade chronic inflammation in many tissues, which is confirmed by increased plasma concentrations of CRP, tumor necrosis factor *α* (TNF-*α*), and interleukins [5, 37]. Bariatric and metabolic surgery shows a positive impact on the reduction of inflammatory biomarkers in several tissues, for example, adipose tissue [3]. CRP, primarily produced in the liver, is known to be upregulated under conditions of obesity, regardless of age, sex, or ethnicity of subjects studied [5]. As a marker of inflammation, plasma concentration of CRP shows a strong and long-lasting decrease after bariatric surgery [38–40]. In the present study, we combined metabolic surgery with a regular and proinflammatory atherogenic diet. After 16 weeks of experimental dietary patterns, DJOS had a strong reductive influence on the CRP plasma level, despite the type of diet used in the experiment, including the HF/HF dietary pattern. A change of the diet from CD to HF after surgery significantly increased CRP plasma levels in SHAM-operated animals when compared to DJOS. The CRP concentrations were consistently lower in DJOS than in SHAM-operated groups, which may be interpreted as a modulation of inflammatory processes even in the conditions of an atherogenic diet. The reduction of plasma CRP was postulated to be associated with weight loss. In the human studies, Selvin et al. observed that for every 1 kg loss of weight in adults, the mean decrease in CRP plasma concentration was 0.13 mg/l, which is probably associated with reduced hypertrophy of adipocytes and lipid storage in adipose tissue [41, 42]. Although, we observed reduced CRP plasma levels after DJOS surgery. Moreover, intragroup-related analyses showed high variations in plasma CRP levels, which resulted in the lack of significant changes between the analysed groups. We hypothesize that it can be explained by the individual responses of the animals. Similar effects were observed in severely obese patients, where their gene polymorphisms were suggested to explain the interindividual variability in circulating CRP [43]. We suggest that a decline in CRP levels may be associated not only with body mass reduction but also with metabolic changes in physiologic profiles of subcutaneous adipose tissue and visceral adipose tissue, lowered adipose inflammation, decreased proinflammatory adipokine production, and lower insulin resistance induced by bariatric surgery [3].

The impaired cross-talk between the endocrine activity of adipose tissue and other insulin-dependent organs is characteristic for obesity and metabolic syndrome [44]. Leptin is the main factor involved in the regulation of energy status, stimulating satiety by metabolic communication between adipose tissue and CNS. This proinflammatory adipokine acts in the early phase of obesity-related inflammation, stimulates proinflammatory immune responses, and plays an important role in energy-deficient states, such as fasting, diet, or exercise-induced amenorrhea and lipoatrophy [3, 45]. To the best of our knowledge, this is the first study that presents the effects of DJOS surgery in combination with different dietary patterns on plasma leptin concentration. DJOS showed a significant impact on leptin plasma levels regardless of the diet applied before and after surgery. We observed a strong effect of DJOS on the leptin plasma levels in all experimental groups when compared to SHAM groups. The lowest level of leptin was observed in the CD/CD dietary pattern in both DJOS and SHAM groups. The highest levels of leptin were detected in conditions of proinflammatory HF/HF dietary intervention before and after surgery. What is interesting is that a change of the diet, in groups where HF was combined with CD, also led to increased serum leptin levels. As previously reported, bariatric surgery-induced weight loss was associated with a positive effect on the endocrine activity of adipose tissue and plasma leptin levels, which decreases independently of the type of surgery performed: Roux-en-Y gastric bypass (RYGB) or laparoscopic sleeve gastrectomy [46]. After RYGB, leptin (protein and mRNA) decreased in patients with diabetes mellitus and dyslipidemia [47]. As an appetite-related hormone, leptin may play an important role in weight regain after obesity therapy. Human studies show significant reduction in leptin blood concentrations in patients who lost at least 5% of body mass using a hypocaloric diet (restriction of 30% of the subject's total energy expenditure) but also a more significant regain of body weight 6 months after the hypocaloric diet in patients who have a higher baseline of fasting leptin levels [48]. In the present study, we demonstrated that HF dietary patterns introduced before or/and after surgery lead to metabolic disturbances, reversing the effects of DJOS and increasing leptin plasma concentration in relation to the control group.

In rodents, chemerin plasma levels were significantly increased in the conditions of dyslipidemia and diminished after fasting [49]. In humans, elevated serum/plasma levels of chemerin are correlated with body fat, glucose, lipid metabolism, and inflammation which is connected with the fact that this adipokine plays a role in the pathophysiology of obesity and metabolic syndrome [50]. Animals fed HF diet showed to be less responsive to chemerin and its physiological actions, such as the regulation of adipogenesis in mature adipocytes, through the activation of chemokine-like receptor 1 (CMKLR1) [51]. Changes in plasma levels of chemerin were reported after biliopancreatic diversion with duodenal switch, which may be associated with improved insulin resistance and resolution from hyperlipidemia [52]. Moreover, changes in plasma chemerin levels have been reported to be time-related and might be a consequence of an improved metabolic phenotype and reduced serum insulin levels. Studying the effects of surgery in relation to eating patterns, a similar trend in leptin and chemerin plasma levels was observed, when comparing the DJOS and SHAM types of surgery. Independently of dietary interventions, there were significantly lower chemerin plasma concentrations after DJOS surgery than in SHAM-operated animals. In addition, we demonstrated that the proinflammatory HF dietary pattern used before or/and after surgery led to an increase in chemerin plasma levels in comparison to CD/CD groups, but did not reduce beneficial effects of DJOS.

## 8. Conclusions

It is concluded that DJOS surgery has a decreasing impact on systemic levels of proinflammatory adipokines and CRP. The beneficial effect of DJOS is strongly deteriorated by an HF diet, in most of the studied combinations, before and/or after surgery. Nevertheless, the beneficial effect of DJOS surgery is stronger than proinflammatory conditions caused by an HF obesogenic diet.

## Figures and Tables

**Table 1:** FABP4, CRP, leptin, and chemerin plasma levels 8 weeks after DJOS (1st column) and SHAM (2nd column) surgery, subjected to 16 weeks of different dietary patterns and intergroup comparison between DJOS and SHAM study groups (3rd column) using descriptive statistics and results of two-way analysis of variance.

Parameter	DJOS				SHAM				*p* ANOVA		
	HF/HF	HF/CD	CD/HF	CD/CD	HF/HF	HF/CD	CD/HF	CD/CD	Group	Op.	Int.
FABP4 (ng/ml)	393.0 ± 46.3	391.8 ± 51.6	397.7 ± 42.1	210.4 ± 66.3	407.1 ± 50.0	428.7 ± 48.1	348.0 ± 31.0	293.4 ± 46.5	**<0.001**	0.133	**<0.05**
CRP (ng/ml)	17.3 (12.2–19.5)	19.4 (18.9–0.4)	11.6 (5.6–14.3)	1.5 (1.1–6.6)	31.9 (31.7–32.2)	27.3 (12.3–28.6)	60.2 (58.8–61.0)	11.0 (2.0–15.6)		**<0.001**	
Leptin (ng/ml)	12.8 ± 3.4	10.4 ± 2.7	10.8 ± 2.6	3.8 ± 0.6	18.3 ± 1.2	17.0 ± 1.6	16.3 ± 2.4	8.3 ± 2.4	**<0.001**	**<0.001**	0.697
Chemerin (ng/ml)	20.6 ± 6.8	15.5 ± 4.1	16.9 ± 4.2	7.8 ± 1.4	35.8 ± 6.7	23.3 ± 2.3	20.1 ± 4.3	15.2 ± 3.5	**<0.001**	**<0.001**	**<0.05**

Statistical significance was set at a *p* < 0.05. FABP4: fatty acid-binding protein 4; CRP: C-reactive protein; DJOS: duodenal-jejunal omega switch surgery; HF: high-fat diet; CD: control diet; HF/HF, CD/HF, HF/CD, CD/CD: type of diet 8 weeks before/8 weeks after surgery; Op.: operation type; Int.: interaction between group and operation type; mean ± standard deviation or median (lower–upper quartile).

**Table 2:** Multiple comparisons in contrast analysis. Column 1: intergroup comparisons between HF/HF, CD/HF, HF/CD, and CD/CD groups DJOS versus SHAM. Column 2: intragroup comparisons between HF/HF, CD/HF, HF/CD, and CD/CD groups after DJOS surgery. Column 3: intragroup comparisons between HF/HF, CD/HF, HF/CD, and CD/CD groups after SHAM surgery.

Post hoc	Column 1: DJOS versus SHAM				Column 2: DJOS						Column 3: SHAM					
	1: HF/HF	2: HF/CD	3: CD/HF	4: CD/CD	1 versus 2	1 versus 3	1 versus 4	2 versus 3	2 versus 4	3 versus 4	1 versus 2	1 versus 3	1 versus 4	2 versus 3	2 versus 4	3 versus 4
FABP4 (ng/ml)	0.609	0.199	0.086	**<0.01**	0.964	0.864	**<0.001**	0.835	**<0.001**	**<0.001**	0.448	**<0.05**	**<0.001**	**<0.01**	**<0.001**	0.052
CRP (ng/ml)	**<0.001**	0.394	**<0.01**	**<0.05**	—	**—**	—	—	**—**	—	—	**—**	—	—	**—**	—
Leptin (ng/ml)	**<0.001**	**<0.001**	**<0.001**	**<0.001**	0.061	0.114	**<0.001**	0.763	**<0.001**	**<0.001**	0.288	0.111	**<0.001**	0.600	**<0.001**	**<0.001**
Chemerin (ng/ml)	**<0.001**	**<0.01**	0.236	**<0.01**	0.052	0.152	**<0.001**	0.605	**<0.01**	**<0.001**	**<0.001**	**<0.001**	**<0.001**	**0.225**	**<0.01**	0.063

Post hoc analysis, statistical significance was set at *p* < 0.05. FABP4: fatty acid-binding protein 4; CRP: C-reactive protein; DJOS: duodenal-jejunal omega switch surgery; HF: high-fat diet; CD: control diet; HF/HF, CD/HF, HF/CD, CD/CD: type of diet 8 weeks before/8 weeks after surgery.
